# Locus Coeruleus magnetic resonance imaging: a comparison between native-space and template-space approach

**DOI:** 10.1007/s00702-022-02486-5

**Published:** 2022-03-20

**Authors:** F. S. Giorgi, N. Martini, F. Lombardo, A. Galgani, L. Bastiani, D. Della Latta, H. Hlavata, C. L. Busceti, F. Biagioni, S. Puglisi-Allegra, N. Pavese, F. Fornai

**Affiliations:** 1 Department of Translational Research and of New Surgical and Medical Technologies, University of Pisa, Pisa, Italy; 2 Deep Health Unit, Fondazione Toscana Gabriele Monasterio, CNR-Regione Toscana, Pisa, Italy; 3 Cardiovascular and Neuroradiological Multimodal Imaging Unit, Fondazione Toscana Gabriele Monasterio, CNR-Regione Toscana, Pisa, Italy; 4 Department of Clinical and Experimental Medicine, University of Pisa, Pisa, Italy; 5 Institute of Clinical Physiology of National Research Council, Pisa, Italy; 6 IRCCS Neuromed, Pozzilli, Italy; 7 Clinical Ageing Research Unit, Newcastle University, Newcastle upon Tyne, UK; 8 Institute of Clinical Medicine, PET Centre, Aarhus University, Aarhus, Denmark

**Keywords:** Locus Coeruleus, Magnetic resonance imaging, Aging, Noradrenaline, Biormarker

## Abstract

Locus Coeruleus (LC) is the main noradrenergic nucleus of the brain, which is involved in many physiological functions including cognition; its impairment may be crucial in the neurobiology of a variety of brain diseases. Locus Coeruleus-Magnetic Resonance Imaging (LC-MRI) allows to identify *in vivo* LC in humans. Thus, a variety of research teams have been using LC-MRI to estimate LC integrity in normal aging and in patients affected by neurodegenerative disorders, where LC integrity my work as a biomarker. A number of variations between LC-MRI studies exist, concerning post-acquisition analysis and whether this had been performed within MRI native space or in *ad hoc*-built MRI template space. Moreover, the reproducibility and reliability of this tool is still to be explored. Therefore, in the present study, we analyzed a group of neurologically healthy, cognitively intact elderly subjects, using both a native space- and a template space-based LC-MRI analysis. We found a good inter-method agreement, particularly considering the LC Contrast Ratio. The template space-based approach provided a higher spatial resolution, lower operator-dependency, and allowed the analysis of LC topography. Our *ad hoc*-developed LC template showed LC morphological data that were in line with templates published very recently. Remarkably, present data significantly overlapped with a recently published LC “metaMask”, that had been obtained by averaging the results of a variety of previous LC-MRI studies. Thus, such a template space-based approach may pave the way to a standardized LC-MRI analysis and to be used in future clinic–anatomical correlations.

**Supplementary Information:**

The online version contains supplementary material available at 10.1007/s00702-022-02486-5.

## Introduction

Locus Coeruleus (LC) is the main noradrenergic (NA) nucleus of the brain and provides the NA innervation for the whole cortical mantle and subcortical structures (Counts and Mufson 2012). LC belongs to the brainstem isodendritic core and is a key element of the so-called reticular ascending activating system (Moruzzi and Magoun 1949; Theofilas et al. 2015). LC contributes to a variety of brain functions, mainly by regulating neuronal homeostasis and modulating neural network activity (Poe et al. 2020). In particular, the LC-NA system plays a pivotal role in neurovascular coupling, blood–brain barrier homeostasis, and microglia modulation (Giorgi et al. 2020a, b); moreover, NA is crucial in wake/sleep cycle, attention and alert systems, and strongly contributes to learning and memory (Poe et al. 2020). This is due to a strong effect on modulating synaptic plasticity (e.g., Giorgi et al. 2006, 2008; Hansen 2017).

In the last decade, several studies have addressed the potential involvement of LC in the pathogenesis of neurodegenerative disorders (Gesi et al. 2000; Kelly et al. 2017). In particular, the development of MRI-based approaches aimed to visualize LC has allowed evaluating its integrity *in vivo* (see the reviews by Galgani et al. 2020; Beardmore et al. 2021). LC Magnetic Resonance Imaging (LC-MRI) has been already used to explore LC involvement in healthy aging (Dahl et al. 2019; Liu et al. 2020; Giorgi et al. 2021), Alzheimer’s Disease (Betts et al. 2019a; Jacobs et al. 2021), Parkinson’s Disease (Sommerauer et al. 2018; Li et al. 2019) and other pathological conditions (reviewed in Galgani et al. 2020). Thus, LC-MRI might add to the large variety of promising novel biomarkers that have been proposed in the last decade to improve diagnostic accuracy in neurodegenerative disorders (Frisoni et al. 2017; Baldacci et al. 2020).

Post-acquisition analysis often represents a major difference among LC-MRI studies; in particular, some authors have profited from data analysis performed in native-space (NS) (Olivieri et al. 2019; Jacobs et al. 2021), while others took advantage of an MRI template-space (TS) *ad hoc* built from their study sample itself (Dahl et al. 2019; Liu et al. 2020).

In a very recent study, Dahl et al. (2021) used a TS-based approach (which requires the construction of an LC mask—see Methods paragraph—identifying the region of interest of the LC in the MRI images), and developed an LC “MetaMask” taking into account a number of previously published LC masks (cited in Dahl et al. 2021). This metaMask was made freely available, thus giving the opportunity also to other groups to test the reliability of their study-specific LC mask and of the MetaMask itself.

In a previous study, we explored the association of LC-MRI features with aging in a group of healthy and cognitively intact elderly subjects, using an NS-based post-processing protocol (Giorgi et al. 2021). Then, in line with the most recent studies on LC-MRI (Dahl et al. 2019), we specifically developed a post-acquisition analysis, building a study-specific LC template, by profiting of those MRI scans acquired in the context of the previous study (Giorgi et al. 2021).

In the present paper, we aimed to assess and discuss the degree of inter-method agreement between the NS- and the TS-based approach. Finally, the LC mask we developed in the present study was compared with the above-described LC metaMask (Dahl et al. 2021).

## Methods

### Characteristics of the study sample

The demographic characteristics and the recruitment protocol of the study sample are reported in detail in Giorgi et al. (2021). Briefly, 53 healthy and cognitively intact elderly subjects (mean age 71.70 ± 4.69 years, 20 males; mean Mini Mental State Examination 27.04 ± 1.27) were recruited at the Pisa University Hospital and Fondazione “G. Monasterio”- CNR/Tuscany Region. Exclusion criteria were: severe medical/cardiological and/or psychiatric comorbidities; neurological disease potentially associated with cognitive decline; history of drugs/alcohol abuse; MRI signs of moderate-severe chronic vascular encephalopathy, according to Fazekas et al. (1987), or other significant alterations. Cognitive and neurological integrity had been evaluated at baseline (T0) and confirmed after 1-year follow-up (T1). Brain MRI was performed within 30 days from T0.

### LC-MRI protocols

MRI scans were performed using a 3T MR-Unit (GE Excite HDx, GE, USA) with an 8-channel phased-array head coil.

**Fig. 1:**
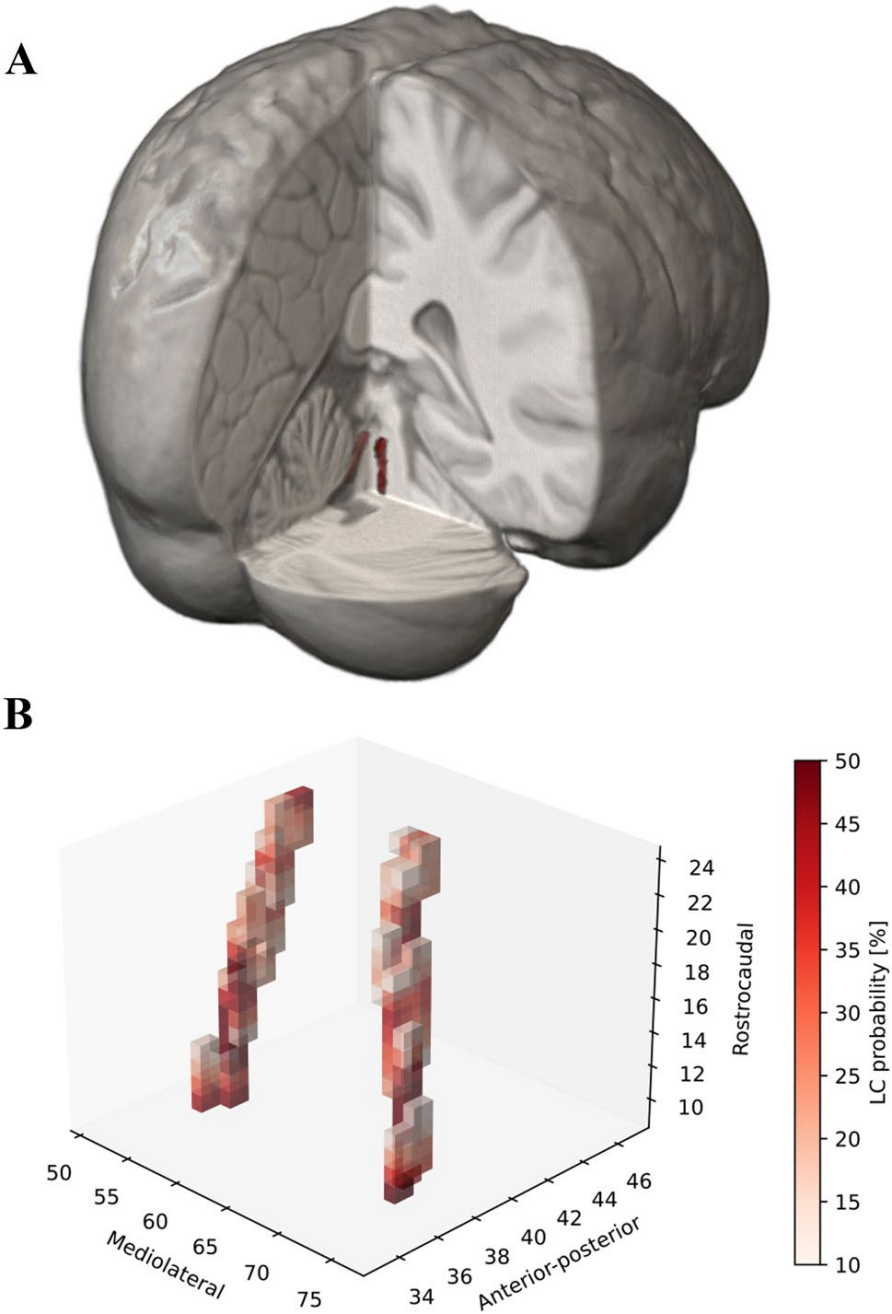
Locus Coeruleus re-construction in MRI space. In panel **A,** it is shown the template-based developed LC mask in the MNI space, reconstructed in 3D. LC extends perpendicularly to the fourth ventricle floor, from the inferior border of the pons to the posterior commissure. In panel **B,** it is reported the probabilistic map of LC-MRI; voxels with a probability higher than 10% to carry LC signal among included subjects are distributed in consecutive MRI slices with a pattern strongly suggestive of LC anatomical counterpart

The LC-sensitive sequence was acquired along the oblique axial plane (Fig. 1A), perpendicular to the fourth ventricle floor, covering an area from the inferior border of the pons to the posterior commissure. We used a 2D-FSE T1-weighted sequence with the following parameters: TR 600 ms; TE 14 ms; flip angle 90°; echo train length 2; NEX 5; matrix size 512 × 384; FOV 200 × 200 mm; pixel size 0.39 × 0.52 mm; 12 contiguous slices, slice thickness 2.2 mm, slice gap 0; acquisition time 14.29 min.

The complete Brain MRI protocol also included 2D-FLAIR, T2* GRE, Spin EchoT1- and FSE T2-weighted with fat saturation and diffusion-weighted imaging sequences. In addition, 3D-Fast-SPGR T1-weighted images were obtained: TR 10.7 ms; TE 4.9 ms; FOV 256 × 256 mm; matrix size 256 × 256; isotropic voxel 1 mm; NEX 1; acquisition time: 5.50 min.

The post-processing imaging analysis provided the quantification of two LC-MRI parameters: LC Contrast Ratio (LC_CR_), which represents the signal intensity of the LC, and the total amount of voxels considered as belonging to the LC (LC_VOX_), which was considered as an indirect estimation of LC volume.

#### Native space-based approach

The semi-automatic NS protocol was already described in Giorgi et al. (2021). Briefly, the analysis was performed by two independent trained operators, using a software developed in-house in Java language. LC region of interest (ROI) was manually placed at the level of the fourth ventricle floor, while two reference ROIs were positioned on the ventral pons (Ref), bilaterally. Once it had been placed in the first slice showing detectable LC-related hyperintensity, the software automatically dragged the ROIs across the contiguous slices. At this stage, the intensity threshold was automatically calculated as the lowest intensity value within the 10 spatially interconnected voxels with maximum intensity (Giorgi et al. 2021). All the voxels exceeding this threshold were highlighted and considered as the first pool of candidate LC-related voxels. The operators evaluated this pool and excluded voxels in anatomically incongruent positions; then, the software re-calculated the threshold and re-screened voxels in the LC-ROI, highlighting possible new candidate voxels. This round was repeated until all suitable voxels were identified. Finally, once the selection stage had been completed, the software computed LC_CR_-NS [LC(intensity)/Ref(intensity)] and LC_VOX_-NS. This method has been set up to provide the values related to both right and left LC combined.

#### Template space-based approach

Our templates for the 3D and the LC-MRI scans were built according to the same procedure proposed by other authors (Dahl et al. 2019), with minor modifications. The workflow steps are detailed below.

##### Whole brain 3D template creation

As the first step, a common space for the 3D anatomical MRI (template_3D_) was created using MRI images of the recruited cognitively intact healthy subjects. These were interpolated to an isotropic resolution of 0.5 mm and a nonuniform intensity correction was applied (*N4 bias field correction*) and then submitted to multi-resolution iterative registration of 3D scans (*antsMultivariateTemplateConstruction2* of the Advanced Normalization Tools v.2.3.4 software (Avants et al. 2011). Registration parameters used were: number of iterations 30 × 90 × 20, cross-correlation similarity metric, Greedy SyN transformation model. Once the registration process had been completed, the aligned 3D volumes were averaged in order to obtain the group whole-brain template_3D_. We also saved the transformation matrices and warping fields that warped the 3D from the individual subject's space to the template_3D_ space (see below).

##### Brainstem template creation

The second step was the creation of a common brainstem space for the acquisition of LC-sensitive sequence. Using the transformations calculated during the whole-brain template_3D_ creation procedure, each scan obtained with LC-sensitive sequence was warped from the native 2D space to the space of the template_3D_ itself. A second multi-resolution iterative registration was run with the same parameters (iterations, similarity metric, transformation model) described above. This spatial registration took into account the anatomical variability between subjects in the LC acquisitions, as well as the possible intra-subject misalignment between the LC and the 3D scans. As a result, we obtained the brainstem template, whose isotropic resolution was 0.5 mm.

##### LC mask creation

In the brainstem template, we found increased intensity and higher signal-to-noise ratio compared to the ventral pons, in a symmetrical bilateral region below the floor of the fourth ventricle. Considering the anatomical location of the signal, the LC/sub-coeruleus complex was considered the main signal source candidate (Betts et al. 2019a; Dahl et al. 2019). To identify those voxels more likely belonging to the LC and, thus, to create the LC mask, a semi-automatic thresholding procedure was performed. An expert operator placed two square 8.5 mm × 8.5 mm reference ROIs bilaterally in the ventral pons in each slice, using ITKsnap (www.itksnap.org) (Yushkevich et al. 2006). The threshold was computed following this formula: μ_ROI_ + 4*σ_ROI_, where μ_ROI_ and σ_ROI_ are the mean value and the standard deviation in the reference ROIs, respectively. Then, a custom program written in Python selected all the voxels exceeding the threshold in the brainstem template, for both hemispheres. These represent the candidate LC-belonging voxels of the brainstem template. The segmentation mask was further refined by an expert neuroradiologist (FL) to exclude voxels that were anatomically incongruent with the LC (e.g., within the fourth ventricle).

##### LC-MRI parameters extraction

Once the LC mask was obtained, LC-sensitive scans of each subject were warped from the native space to the brainstem template space for data analysis, and the parameters LC_CR_ developed by TS-based approach (LC_CR_-TS) and LC_VOX_ developed by TS-based approach (LC_VOX_-TS) were extracted. The parameter LC_CR_-TS was calculated using the following formula: LC_C*R = *_[max(LC)—max(Ref)] / max(Ref), where max(Ref) and max(LC) are the maximum signal intensities in the left and right reference regions, and LC regions derived from the LC template segmentation, respectively.

LC_VOX_-TS was calculated as follows. First, the LC mask in the brainstem template was considered as the search space of the LC voxels. Then, reference ROIs were drawn on the LC scan warped to the brainstem template, and a subject-specific threshold was calculated using the same formula used for the LC mask. The number of voxels in the search space that exceed the subject-specific threshold constituted LC_VOX_-TS parameter value.

For each subject, the two LC-MRI parameters (LC_CR_ and LC_VOX_) were calculated for left and right hemisphere separately (“left LC” and “right LC”, respectively), or for left and right LC combined (“combined LC”).

In order to maximally reduce any potential side-related intensity artifacts which might affect the results, we applied to LC scans a field correction algorithm (Tustison et al. 2010), and LC-related parameters were standardized using bilateral ROIs as reference regions, placed symmetrically in the left and right ventral pons (see above).

### Comparison with previously published metaMask

Recently, an LC mask obtained by pooling a variety of study-specific LC masks (“metaMask”), was released and made freely available (https://osf.io/sf2ky/) (Dahl et al. 2021). To assess the compatibility between our TS-based approach and the published metaMask, we evaluated the overlap in the MNI space between our LC mask and the metaMask. To warp our LC mask into the MNI space, we first co-registered the brainstem template to the 0.5-mm iso-voxel MNI template using linear (rigid, then affine), followed by nonlinear (SyN) registration (Avants et al. 2011). Then, these transformations were applied to the LC mask using a nearest-neighbor interpolation to warp the LC mask into the MNI space. The degree of compatibility between our LC mask and the metaMask was expressed using the same parameters that Dahl et al. themselves described in their study, namely specificity and sensitivity (Dahl et al. 2021). These were calculated as the ratio between the number of voxels the study-specific LC mask and the LC metaMask have in common and the total number of voxels of the former and the latter, respectively. We calculated also the “accuracy” which was defined as the mean between sensitivity and specificity of the mask (Dahl et al. 2021). For comparison, we also warped the metaMask into the brainstem template through inverse transformation, and we additionally calculated the LC parameters using the metaMask in the TS approach.

### Statistical analysis

Data normality was assessed through Shapiro–Wilk test and visual checking of heteroskedasticity. Since they did not show a Gaussian distribution, variables were Z-standardized. Pearson’s correlation was used to evaluate inter-method agreement (van Stralen et al. 2008) and age effect on TS-based LC parameters. A Paired-sample t-test was used to assess side-related differences within TS-based LC variables. Multiple comparisons were adjusted with False Discovery Rate (FDR) correction to rule out type I error. The level of significance was set at *p* < 0.05. All statistical analyses were performed using SPSS Version 25. GraphPad Prism 8.0 was used for graphical representations.

## Results

### Template space-based LC reconstruction

The spatial co-registration of each LC-sensitive scan to the brainstem template was carefully visually inspected. The obtained brainstem template showed marked hyperintensities bilaterally in the LC region, while the region of the ventral pons showed a rather homogeneous distribution. The final LC mask included a total number of 234 voxels across 17 axial slices. Figure 1B shows the 3D visualization of the LC probabilistic map, reflecting the coordinates of the maximum value of the left and right LC for each slice.

### Template space-based LC features

We did not find any effect of age on TS-based LC parameters, neither considering the combined LC (LC_CR_-TS, *r = *-0.291, *p = *0.07, LC_VOX_-TS, *r = *-0.254, *p = *0.066), nor analyzing the two LC separately (LC_CR_-TS, *r = *− 0.213, *p = *0.167, LC_VOX_-TS, *r = *− 0.273, *p = *0.096 for the left LC; LC_CR_-TS, *r = *− 0.322, *p = *0.076, LC_VOX_-TS, *r = *− 0.254, *p = *0.152, for the right LC).

**Fig. 2:**
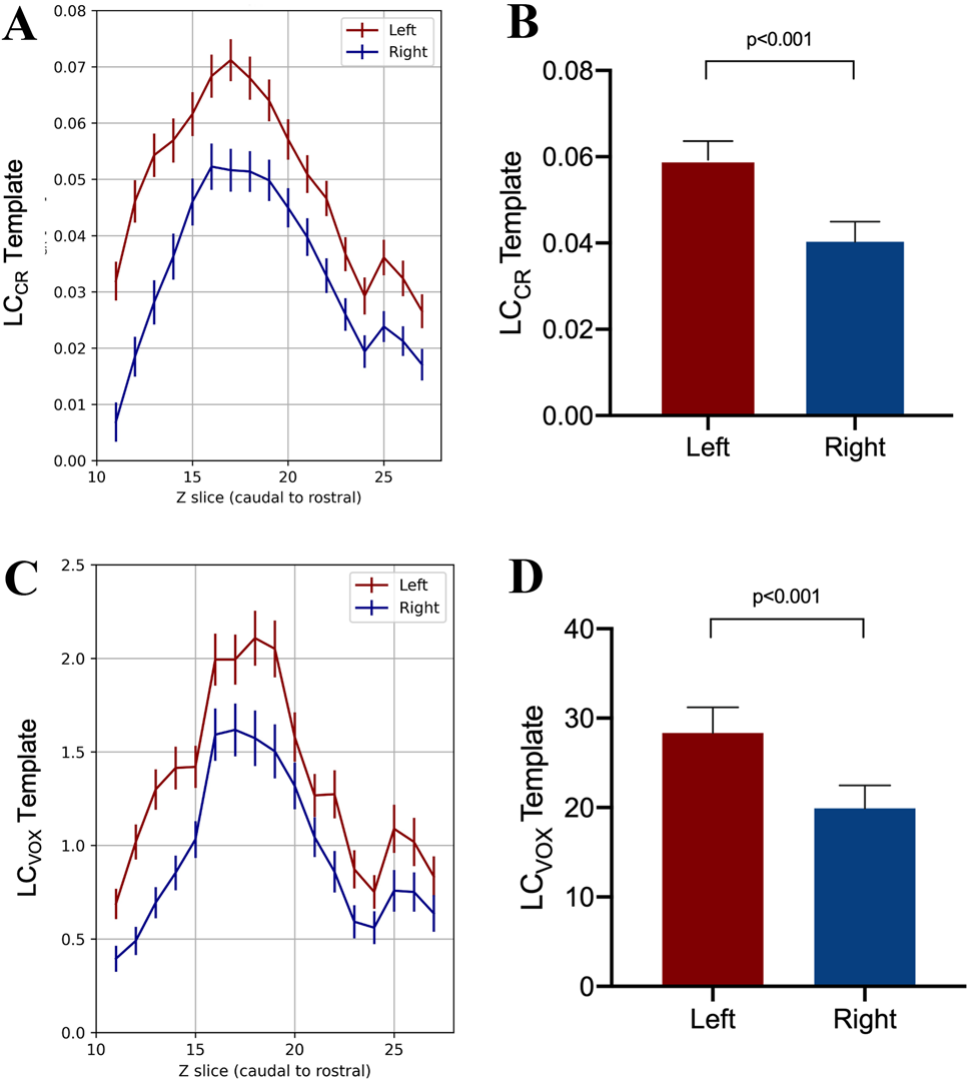
Side-related LC differences. The figure reports the results of LC analysis of the two LC separately, with the TS-based approach. The left LC showed higher values of LC_CR_ (**B**) and LC_VOX_ (**D**) when compared to the right one. Such a characteristic was homogeneously present through the whole LC rostral–caudal extension (**A**, **C**). LC_CR_ and LC_VOX_ are plotted as mean values ± SEM

A side-related difference was observed between the two LCs. LC_CR_-TS and LC_VOX_-TS values of the left LC were higher than those of the right LC (*t* = 5.556, df = 52, *p* < 0.001; *t* = 4.434, df = 52, *p* < 0.001, respectively) (Fig. 2). Remarkably, there was no significant difference between left and right reference regions intensity values (*t* = 0.518, df = 52, *p = *0.607).

### Inter-method agreement

**Fig. 3:**
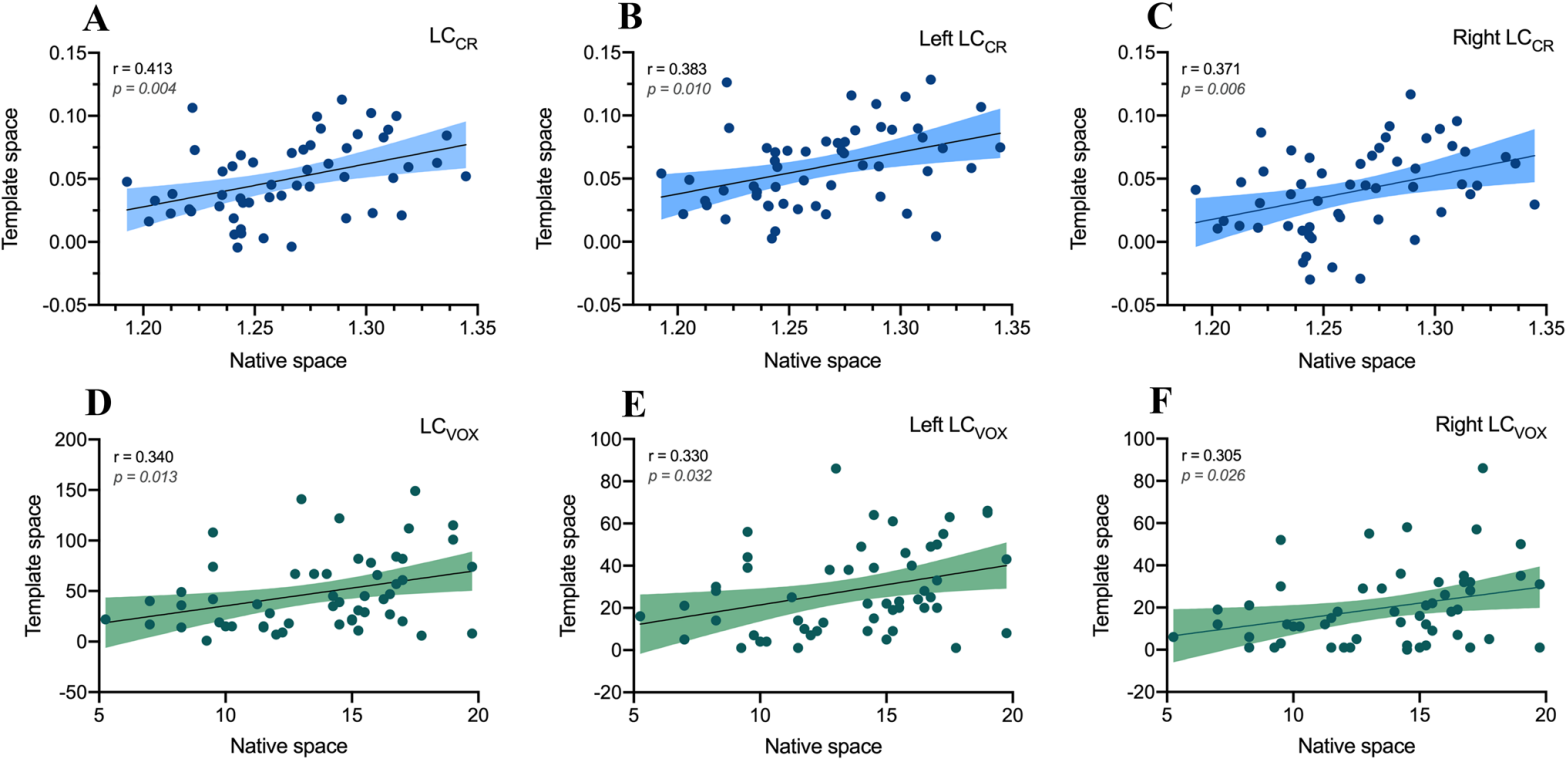
Inter-method agreement. The figure shows the scatterplots of LC-MRI parameters computed either with the template-space method (Y-axis) or the native-space one (X-axis). The Pearson’s correlations coefficient (r) was calculated for both LC_CR_ (panels **A**, **B**, **C**) and LC_VOX_ (**D**, **E**, **F**) of the combined LC (**A**, **D**) and the left (**B**, **E**) and right (**C**, **F**) LC separated. Shaded colors represent the 95% confidence intervals. Reported p-values were adjusted for multiple comparisons with FDR correction

There was a good agreement between the signal intensity measures obtained by the two methods. LC_CR_-NS showed a direct correlation with LC_CR_-TS values obtained from two LC combined (*r = *0.413, *p = *0.004) (Fig. 3A). It was evaluated also the correlation of left and right LC values, separately assessed with the TS-based approach, with the single (i.e., including the values from left and right LC) parameters extracted with the NS-based approach, in order to assess whether the latter ones were more related to the LC signal of one side or the other one. We found that the same trend described above for the combined LC was observed when left and right LC were analyzed separately (*r = *0.383, *p = *0.010 and *r = *0.371, *p = *0.006, respectively) (Fig. 3B and C).

The NS- and TS-based methods showed a significant agreement with each other also concerning volume-related measures (although the inter-method agreement was lower compared to the LC_CR_ ones), when considering either the combined LC (*r = *0.340, *p = *0.013) (Fig. 3D) or the two LC separately (*r = *0.330, p 0.032 for the left LC_VOX_, *r = *0.305, *p = *0.026 for the right LC_VOX_) (Fig. 3E and F).

### Compatibility of the template-based approach with LC metaMask

**Fig. 4:**
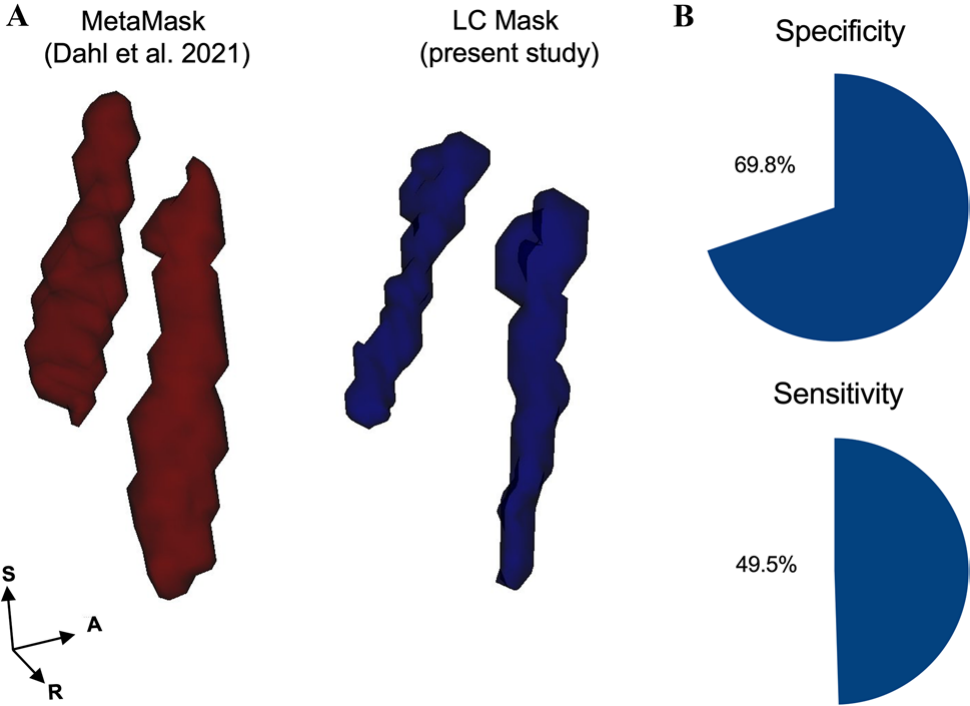
Compatibility of our LC mask and the LC metaMask. Panel **A** shows the 3D rendering of our LC mask and published LC metaMask (Dahl et al. 2021) in the MNI space. Panel **B** reports pie charts representing sensitivity and specificity of our LC mask (sensitivity: number of voxels the LC mask and the LC metaMask share with each other/total number of voxels of the metaMask; specificity: number of voxels the LC mask and the LC metaMask share with each other/total number of voxels of the LC mask—according to definitions provided in Dahl et al. 2021)

We found a high degree of compatibility between our LC mask and the LC metaMask (specificity 69.8%, sensibility 49.5% and accuracy 59.6%; Fig. 4). Moreover, the LC-MRI parameters calculated using our LC mask showed very strong direct correlation coefficients with those calculated using the LC metaMask (Supplementary Table 1).

## Discussion

*In vivo* LC analysis by MRI has been performed by several authors and it will likely be a matter of interest also in future years, as LC degeneration represents a promising marker in a number of neurodegenerative disorders (Betts et al. 2019a; Galgani et al. 2020). In this study, we presented the results of an LC-MRI analysis performed using both an NS- and a TS-based approach in a group of cognitively healthy subjects. We successfully built an LC-MRI template and used it to extract LC imaging parameters. Then, we compared the results obtained with both approaches, and we showed a good inter-method agreement for LC_VOX_, and mostly for LC_CR_.

LC_CR_ appears to be more directly related to LC features, since it simply represents the ratio between the MRI signal intensity of LC and the reference regions. This might explain why LC_CR_ showed a higher correlation coefficient between the two methods. Nonetheless, there was not a complete concordance between NS- and TS-based approaches regarding this parameter. This may depend on the higher spatial resolution of the TS-based approach, which allows the identification of many voxels that were potentially excluded from the NS-based analysis, thereby affecting LC_CR_ values.

Such a higher spatial resolution may also explain the lower concordance between NS- and TS-based approaches concerning the parameter LC_VOX_. Thus, LC_VOX_ may be useful for LC-MRI analysis studies, since it provides the estimation of authentic LC volume. Even though we have already tried to extrapolate this parameter also using the NS-based approach (Giorgi et al. 2021), LC_VOX_ computed with the TS-based method might be more appropriate to calculate the LC anatomical volume. In fact, as shown in present paper, and in line with what already shown by the groups that used a similar approach (e.g., Betts et al. 2017; Dahl et al. 2019), the spatial distribution of LC voxels closely followed LC anatomy. Concerning this aspect, it is also worth emphasizing that the distribution of LC_VOX_ showed a higher value in the middle part of the LC mask and a lower one in its rostral and caudal parts; this is in line with anatomical data, which repeatedly showed that LC neurons are more abundant and densely packed in the middle part of the nucleus, while they are scattered towards LC rostral and caudal poles (Schwarz and Luo 2015). Also, the length and the volume of the nucleus estimated from the LC mask were close to what measured in histological studies (Fernandes et al. 2012) (Fig. 1A).

In a previous paper, in which we profited from the NS-based approach only (Giorgi et al. 2021), we did not find any association between LC features and age in this group of subjects. Here, we confirmed this observation also with the TS-based approach, both when merging data from right and left LC, and when considering the left and right LC separately. These findings are in line with recent *post-mortem* data, which, by profiting from unbiased stereology, did not show any significant LC involvement during physiological aging (Theofilas et al. 2017).

The left/right side-related difference we observed in the distribution of LC-MRI parameters deserves a special emphasis. Left LC showed higher LC_CR_ and LC_VOX_ values compared to the right one. To our knowledge, this has never been specifically addressed in post-mortem studies of LC. MRI studies of LC carried out by other authors (Betts et al. 2017; Liu et al. 2019) are compatible with the present finding in spite of different MRI scanners and LC post-acquisition methods. In the present study, all efforts were made to avoid any potential machine-related influence on LC signal (as witnessed also by the lack of differences between left and right ventral pons reference ROIs intensity), nonetheless side-related difference may still be partly due to uncommon artifacts (see also Betts et al. 2017). Such an asymmetry needs to be validated and, when confirmed, it should be specifically analyzed to better understand the role of LC in brain activities and brain disorders.

So far, all research teams that profited from a TS-based approach developed specific LC mask based on their own subjects’ sample (e.g., Betts et al. 2017; Dahl et al. 2019). However, in a very recent study Dahl et al. (2021) developed a so-called LC metaMask. This was built-up by pooling together the LC masks developed by various research groups. Concordance of the metaMask with each specific study-dependent mask was assessed in the same study (Dahl et al. 2021). The fact that our LC mask remarkably overlaps with Dahl’s group’s metaMask, even though it was not used to build it, lends further substance to the reliability of the TS-based approach for LC imaging studies.

In conclusion, our study showed a good agreement between NS- and TS-based approaches, with the latter method offering more advantages in terms of spatial resolution and regional analysis of LC. Furthermore, since the TS-based approach drastically reduces operator-dependency, it would pave the way to a standardized methodological protocol for LC-MRI analysis and, thus, to its possible clinical application. As already mentioned, LC has been receiving growing attention in recent years for its possible role both in the pathogenesis and pathophysiology of Alzheimer’s Disease (Rorabaugh et al. 2017; Weinshenker et al., 2018), and thus it might even become a target of potential disease-modifying drugs. In such a context, LC-MRI may be used in the future to identify the Alzheimer’s Disease patient phenotype with the highest burden of LC pathology and more likely to benefit from a LC-targeted therapy.

## Supplementary Information

Below is the link to the electronic supplementary material.

Supplementary file1 (DOCX 14 KB)
