# Supplementary material for: Locus Coeruleus magnetic resonance imaging: a comparison between native-space and template-space approach
Source: J Neural Transm (Vienna). 2022 Mar 20;129(4):387–94. doi: 10.1007/s00702-022-02486-5 (PMC9007774; doi:10.1007/s00702-022-02486-5)
Supplement: Supplementary file 1 — Supplementary file1 (DOCX 14 KB) [file 702_2022_2486_MOESM1_ESM.docx]

**Supplementary Table 1. LC_CR_ and LC_VOX_ parameters calculated in template space using our LC mask and the LC metaMask**

| **LC-MRI parameter** | | **r** | ***p value*** |
| --- | --- | --- | --- |
| **LC_CR_** | *Combined LC* | 0.998 | <0.001 |
|  | *Left LC* | 0.998 | <0.001 |
|  | *Right LC* | 0.997 | <0.001 |
| **LC_VOX_** | *Combined LC* | 0.993 | <0.001 |
|  | *Left LC* | 0.982 | <0.001 |
|  | *Right LC* | 0.986 | <0.001 |

LC-MRI parameters were calculated on the brainstem template space using both our mask and the published LC metaMask (Dahl et al. 2021). The values obtained were compared using Pearson’s correlation test. All the examined parameters showed very strong direct correlation coefficients with each other. LC_CR_: Locus Coeruleus Contrast-Ratio; LC_VOX_: Locus Coeruleus number of voxels; r: Pearson’s coefficient. P-values are adjusted con FDR correction for multiple comparisons.
